# Cloning, characterisation and comparative analysis of a starch synthase IV gene in wheat: functional and evolutionary implications

**DOI:** 10.1186/1471-2229-8-98

**Published:** 2008-09-30

**Authors:** Marina Leterrier, Lynn D Holappa, Karen E Broglie, Diane M Beckles

**Affiliations:** 1 Department of Plant Sciences, One Peter Shield Avenue, University of California, Davis, CA 95616-8617, USA; 2 DuPont-Pioneer, Crop Genetics Research, Experimental Station, Wilmington, DE 19808, USA; 3 Department of Organismic & Evolutionary Biology, Harvard University, 16 Divinity Ave, Cambridge MA 02138, USA

## Abstract

**Background:**

Starch is of great importance to humans as a food and biomaterial, and the amount and structure of starch made in plants is determined in part by starch synthase (SS) activity. Five SS isoforms, SSI, II, III, IV and Granule Bound SSI, have been identified, each with a unique catalytic role in starch synthesis. The basic mode of action of SSs is known; however our knowledge of several aspects of SS enzymology at the structural and mechanistic level is incomplete. To gain a better understanding of the differences in SS sequences that underscore their specificity, the previously uncharacterised *SSIVb *from wheat was cloned and extensive bioinformatics analyses of this and other SSs sequences were done.

**Results:**

The wheat SSIV cDNA is most similar to rice *SSIVb *with which it shows synteny and shares a similar exon-intron arrangement. The wheat *SSIVb *gene was preferentially expressed in leaf and was not regulated by a circadian clock. Phylogenetic analysis showed that in plants, SSIV is closely related to SSIII, while SSI, SSII and Granule Bound SSI clustered together and distinctions between the two groups can be made at the genetic level and included chromosomal location and intron conservation. Further, identified differences at the amino acid level in their glycosyltransferase domains, predicted secondary structures, global conformations and conserved residues might be indicative of intragroup functional associations.

**Conclusion:**

Based on bioinformatics analysis of the catalytic region of 36 SSs and 3 glycogen synthases (GSs), it is suggested that the valine residue in the highly conserved K-X-G-G-L motif in SSIII and SSIV may be a determining feature of primer specificity of these SSs as compared to GBSSI, SSI and SSII. In GBSSI, the Ile485 residue may partially explain that enzyme's unique catalytic features. The flexible 380s Loop in the starch catalytic domain may be important in defining the specificity of action for each different SS and the G-X-G in motif VI could define SSIV and SSIII action particularly.

## Background

Starch is an important reserve of carbon and energy. It is the most widely used storage compound in plants and it makes up 50% of the calories humans eat [1]. Further, starch has great utility in several industries as a cheap, versatile and biodegradable polymer and its demand in these niche markets is increasing [2,3]. In spite of its importance as a primary source of food for humans and its growing economic value as a raw material, our understanding of the mechanistic basis of starch biosynthesis is incomplete [1,4,5].

Starch is deposited in the plastid as complex crystalline granules. It is made up of two α1–4-glucan polymers; amylose is the smaller almost linear polymer, while amylopectin is larger and has a high frequency of α-1–6-glycosidic linkages between glucan chains [2]. The branch pattern of α-1–6-linked amylopectin glucan chains is polymodal and highly organised such that crystallisation of glucose molecules occurs, with the subsequent formation of a granular supra-macromolecule. Prokaryotes, fungi and animals do not store starch but accumulate glycogen. Although glycogen also has branched α-1,4-glucan chains, they lack the sophisticated polymodal organisation found in starch. The result is that glycogen is amorphous and water-soluble [6], while starch is a metabolically stable hydrocolloid [7,8].

Starch synthases (SSs; EC 2.4.1.21 and EC 2.4.1.242) elongate the glucan chains of amylose and amylopectin. They catalyze the transfer of the glucose moiety of ADPglucose to the non-reducing end of an existing glucan chain via an α-1,4-glucosidic link. Five SS classes, SSI, SSII, SSIII, SSIV and Granule Bound SSI (GBSSI), have been identified among the DNA sequences available in public repositories and, in cereals, duplication of several SS-encoding genes gave rise to 10 isoforms [9]. Genome sequencing projects of unicellular green algae show that most of the SS classes found in higher plants are also present in algae [6], suggesting that the gene duplication events that gave rise to the five main SS isoforms predated the evolution of higher plants. Prokaryotic glycogen synthases (EC 2.4.1.11; GS), like higher plant SSs, use the ADPglucose pathway for the synthesis of glycogen; however, surveying the genomic sequences of prokaryotes in DNA databanks leads to the conclusion that there may be no more than two GS isoforms in prokaryotes.

The coordinated activity of the different SS isoforms helps to build the starch granule and influence its architecture [8,10]. Genetic, and some biochemical and *in vitro *kinetic studies, suggests that SSI elongates the short chains, SSII the intermediate, and SSIII and perhaps GBSSI elongate the long chains of amylopectin, respectively [8]. Only GBSSI activity is required to make amylose [10]. A role for the SSIV isoform primarily in starch granule initiation in *Arabidopsis *has recently been shown although it may also be involved in making short glucan chains [11]. While it is largely held that each SS isoform has a unique role in starch biosynthesis, studies of some SS transgenic and mutant lines suggest that there may be functional overlap between isoforms [11-13]. In addition to chain-length preferences, SS isoforms also have distinct intra-plastidic locations. GBSSI is starch granule-localised, SSIII and SSIV [11] are exclusively in the stroma, while SSII and SSI are found distributed between stroma and granule. The partitioning of SS activity in these different regions may influence their individual kinetic behaviours through their interaction with substrate and other starch biosynthetic enzymes, with eventual repercussions for granule structure.

The basic metabolic activity of SSs and GSs is defined by a highly conserved region shared between these enzymes. This "core" region is in the C-terminus of plant SSs and is 60 kDa in length, but encompasses essentially the entire protein sequence of the prokaryotic GSs. The major variation among SSs in this region is an extension or "tail" of 20 amino acids found only in GBSSI proteins [14]. Within the catalytic core is the conserved starch catalytic domain (Pfam PF08323) and the glycosyltransferase 1 domain (Pfam PF00534) characteristic of the GT5 glycosyltransferase superfamily according to CAZY [15-18]. The starch catalytic domain is found in glycosyltransferases that use only ADPglucose as substrate. The glycosyl transferase 1 domain is found in proteins that transfer UDP, ADP, GDP or CMP-linked sugars to a variety of substrates, including glycogen, fructose-6-phosphate and lipopolysaccharides and is therefore not unique to GSs and SSs.

Our knowledge of how GT5 enzymes interact with the ADPglucose substrate was enhanced by the determination of the crystal structure of *Agrobacterium tumefaciens *GS and modeling of its interaction with substrate [16,19]. *Agrobacterium tumefaciens *GS and perhaps all SSs and GSs are "retaining-type" glycosyltransferases with a GT-B-fold [18,19]. The GT-B-fold is characterised by two different Rossmann-like α-β-α domains, separated by a deep cleft that is predicted to be highly flexible [16,19]. Within this fold is one of the two highly conserved K-X-G-G-L motifs that may play a role in catalysis and/or substrate binding [20-25]. Although there are no SS crystals available to enable definitive conclusions about SS three-dimensional (3D) structure, the sequence homology of bacterial GS and the catalytic core region of the plants makes it likely that the structural features described for *Agrobacterium tumefaciens *GS may also hold true for plant SSs [26].

In contrast to the similarities at the C-termini, the N-terminal region upstream of the catalytic core, shows no sequence uniformity amongst SS isoforms. Further, SS N-termini vary greatly in length from 2.2 kDa in GBSSI to ~135 kDa in maize SSIII [27], and noticeably, is absent in prokaryotic GSs. The SS N-terminal extension does not appear to be directly involved in catalysis although it may alter SS kinetics and/or interaction with substrate [12,24,28]. It could also play a crucial role in the ability of different SSs to form functional enzymatic complexes with other starch biosynthetic enzymes, which is proving to be key factor in determining amylopectin formation [29,30].

This paper describes the cloning of a cDNA from wheat with high homology to the SSIV class of SSs, and the bioinformatics analysis of this and other SSs sequences in the database to investigate potential functional, structural and evolutionary relationships between SSIV, other SSs and GSs. The first aim was to gain a basic understanding of the *SSIV *gene in wheat – its expression, genomic organisation and chromosomal location. The second aim was to identify similarities in the predicted protein sequences of GSs and SSs that may indicate important conserved characteristics critical to SS function. The third aim was to identify sequence differences that may denote evolutionary changes that serve as the basis for the specificity of action of these SS isoforms.

In this manuscript the different SS genes and their predicted proteins are described as follows: the prefix refers to the species name, e.g. *Triticum aestivum *L. is referred to as *Ta*, and is followed by indication of the SS isoform, e.g. SSIV. For duplicated SS genes in cereals, we use the suffix *a*, *b *or *c *to indicate that sub-isoform, depending on the organ where the gene is primarily expressed; *a *refers to storage tissue and *b *and *c *to vegetative tissue. For consistency GBSSI and GBSSII are described as GBSSIa and GBSSIb, respectively, as originally named by Denyer *et al*. [31].

## Results

### A. Cloning and characterisation of SSIVb

#### Identification and characterization of a wheat SSIV cDNA

**Figure 1:**
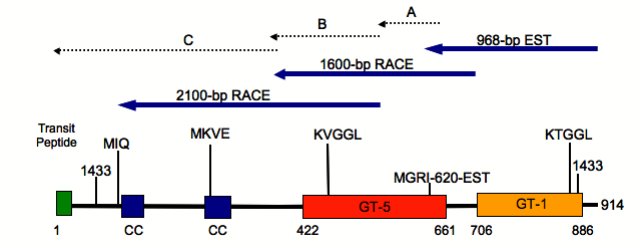
**A schematic diagram showing the PCR fragments that make up the contiguous *SSIV *cDNA as well as domains found within the predicted amino acid sequence**. PCR fragments A, B and C were amplified by a 5'RACE-like procedure carried out with wheat cDNA libraries (see Methods) and represent the minimum tiling path of several sequences identified. The SSIV EST (EST-968 bp) and the cDNAs called 1600 bp RACE and 2100 bp RACE were isolated similarly to A, B and C and were assembled into different cloning vectors to test coding potential and/or glycosyltransferase activity. The starch catalytic domain (GT-5) and glycosyltranferase domain (GT-1) characteristics of the SS family are shown. Predicted 14-3-3 recognition sites and the coiled-coil domains (blue boxes and 'CC'), as well as the two highly conserved KVGGL and KTGGL domains are also shown.

An expressed sequence tag (EST) [Genbank:BT009276] homologous to plant SSs and glycogen GSs at the amino acid level was identified from a developing wheat seedling cDNA. Using a 5'RACE-like procedure, contiguous cDNA fragments were amplified from wheat libraries that collectively gave a 3386 bps-long sequence containing a full-length ORF of 2806 bp [Genbank:AY044844] (Figure 1). The amino acid sequence deduced from this ORF contains 914 residues and a predicted molecular mass of 103.1 kDa. A putative cleavage site between amino acids 45 and 46 was identified using the ChloroP neural network [32] cleavage at which would result in a mature protein of molecular mass 98.3 kDa. Analysis of the sequence showed that it was most homologous (87%) to *Os*SSIVb [Genbank:AAQ82623].

The SSIV cDNA sequence allows it to be unambiguously identified as a SS. The starch catalytic and glycosyltransferase domains characteristic of SSs are present in the C-terminus (Figure 1), and conservation of sequence identities at the two putative ADPglucose binding motifs, i.e. KVGGL and KTGGL, was found between this wheat cDNA and the rice SSIV and SSIII Classes. Expression of the 968-bp EST and 2100-bp RACE products in *E. coli *produced polypeptides of the expected size. In the latter case, glycosyltransferase activity could be detected, albeit at a low level [see Additional file 1]. Like all other SSs the N-terminus of *Ta*SSIVb is unique; an SSIV-specific region, from amino acids 1–405, was detected and within this region two coiled-coil domains and a 14-3-3-protein recognition site were identified (Figure 1).

#### Phylogenetic analysis of starch and glycogen synthases

**Figure 2:**
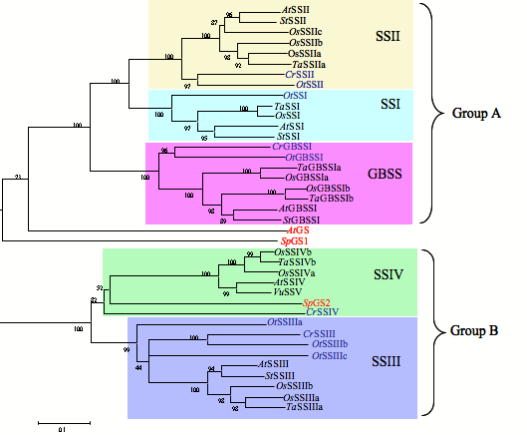
**Phylogenetic tree showing the relationship between plant and algal SS and prokaryotic GS isoforms on the basis of predicted amino acid sequence**. The tree was constructed using the neighbour-joining method and confidence limits to branch points in the tree were assigned by bootstrapping the alignment with a random number generator seed of 111 and 1000 trials. Unicellular algae SSs are shown in blue and GSs are in red. Each node is labeled with the prefix of the initials of the genus and species, e.g. *At *= *Arabidopsis thaliana Agt *= *Agrobacterium tumefascien*; *Cr *= *Chlamydomonas reinhardti*; *Sp *= *Synechocystis*; *Os *= *Oryza sativa*;, *Ot *= *Ostreococcus tauri*; *St *= *Solanum tubersum*; *Ta *= *Triticum aestivum*; and *Vu *= *Vignia unguiculata*; Genbank accession numbers of the starch synthases and glycogen synthases shown in the tree are as follows: *At*SSI [AAF24126], *At*SSII [AAF26156]*At*SSIII [AAD30251], *At*SSIV [CAA16796], *At*GBSSI [NP_174566], *Cr*GBSSI [AAL28128], *Cr*SSII [AAC17970], *Cr*SSIII [AAY42381], *Cr*SSIV [AAC17971], *Agt*GS [AAD03474], *Os*GBSSI [AAC61675], *Os*SSI [AAL16661], *Os*SSIIa [Q5DWW9], *Os*SSIIb [AAK81729], *Os*SSIIc [AAK64284], *Os*SSIIIa [AAM49811], OsSSIIIb [AAL40942], *Os*SSIVa [AAQ82622], *Os*SSIVb [AAQ82623], *Os*GBSSI [AAF72562], *Os*GBSSIb (called GBSSII [BAF26592]) *Sp*GS-1 [P74521], *Sp*GS-2 [P72623], *St*SSI [P93568], *St*SSIII [T07663], *Ta*GBSSI [BAA77351], *Ta*SSI [CAB99209], *Ta*SSIIa [CAB86618], *Ta*SSIIIa [AAF87999], *Ta*SSIIIb [ABY56823]*Ta*SSIV [AAK97773], *Vu*SSIII [CAB40374], *Vu*SSV [CAB40375], *Ta*GBSSII [AAF14233], *Ot*SSI [CAL56451], *Ot*SSII [CAL58277], *Ot*SSIIIa [AAS88893], *Ot*SSIIIb [AAS88894] and *Ot*SSIIIc [AAS88881].

The deduced amino acid sequence of the wheat SSIV cDNA was compared to those of 36 other SSs and 3 GSs, two from *Synechocystis *and the other from *Agrobacterium tumefaciens*, using Clustal W [33,34]. The five SS classes clustered into two groups (Figure 2), an arrangement also reported by others [6,35]. For ease of description, GBSSI, SSI and SSII will be called the Group A SSs, and SSIII and SSIV will be called the Group B SSs, respectively. Within the Group A SSs, GBSSI appears to diverge from SSI and SSII. *Ostreococcus *does not have a SSIV gene but has three isoforms of SSIII, with the *Ot*SSIIIa most closely resembling other SSIV proteins. *Synechocystis *PCC 6803 is one of the few cyanobacteria identified from BLAST analysis to have two GS isoforms, one of which repeatedly clustered with the Group B SSs, and the other repeatedly with the Group A SSs. This was found in all phylogenetic trees created whether using neighbour-joining, minimum evolution or maximum parsimony on both nucleotide and protein sequences.

#### Genomic organisation of *TaSSIVb*

**Figure 3:**
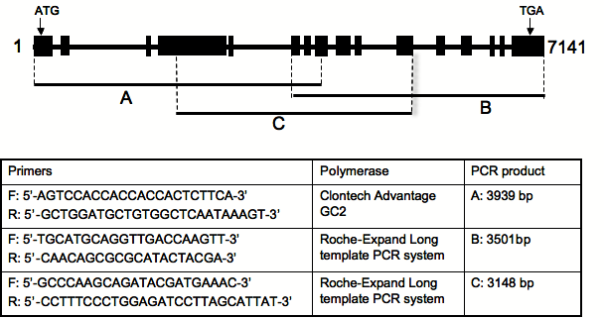
**Partial genomic sequence of *TaSSIVb *showing the exon-intron arrangement**. The *TaSSIVb *genomic clone was isolated by PCR using genomic DNA from a *Triticum aestivum *cv. Chinese Spring line nullisomic for chromosome 1B and tetrasomic for chromosome 1D as template. The primers used for amplification are indicated in the box and were designed from the cDNA sequence. The longest PCR fragments (A, B and C) that gave the minimum tiling path are indicated. Exon-intron organisation of the assembled genomic sequence was predicted by Spidey [75], which compares cDNA against genomic DNA sequence. Exons are indicated by the black boxes.

A partial genomic clone of SSIV [Genbank:DQ400416)] was sequenced from *Triticum aestivum *using PCR. Three overlapping contiguous genomic clones (See Figure 3) together gave a sequence of 7,141 bp. The promoter region could not be amplified in our hands presumably because of the high GC content of the 5' end of the gene. The *TaSSIVb *gene contains 16 exons separated by 15 introns, a structure which is similar to that reported in all SS isoforms cloned to date [36]. Although intronic sequences are generally only weakly similar among plant SSs, there is high identity (ranging from 51.1 to 78.3%) between introns four to nine of *TaSSIVb *and *OsSSIVb *while the identity between the introns of the two *OsSSIV *isoforms averaged only 20% (data not shown). Of note is the similarity of sequence found in intron-4 among all the SSIV isoforms from wheat, rice and *Arabidopsis *(40 to 47%).

#### Chromosomal location of *TaSSIVb*

**Figure 4:**
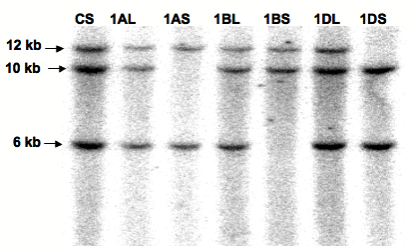
**Chromosomal Mapping of *TaSSIVb *by RFLP analysis of Chinese Spring ditelosomic lines**. Approximately 20 μg of wheat genomic DNA was digested with *EcoRV *and blotted onto HyBond N+, and was hybridised against a 550 bp fragment from a *Pst*I/*EcoR*I digest of clone 1600 bp RACE (Figure 1). The estimated size of each band is indicated on the right. The nomenclature for each sample is as follows: CS, Chinese Spring parental; Ditelosomic lines, 1AL, 1BL and 1DL, lack the short arms of chromosomes 1A, 1B and 1D, respectively, but each has four copies of the corresponding long arms. Ditelosomic lines, 1AS, 1BS and 1DS, lack the long arms of chromosome 1A, 1B and 1D, respectively, but each has four copies of the corresponding short arm. Bands missing in 1AS, 1BS and 1DS show that SSIV is likely located on the long arm of Chromosome I.

The chromosomal location of the *TaSSIVb *gene was determined by Southern blot analysis of wheat nullisomic-tetrasomic and ditelosomic lines [37]. Three bands of approximately 12 kb, 10 kb and 6 kb hybridized to the SSIV probe (data not shown) in nullisomic-tetrasomic lines, indicating that *TaSSIVb *is a single-copy gene, one gene present on the homeologous group I chromosome of each of the A, B, and D genomes. Additional mapping experiments with ditelosomic lines showed that the 10 kb band was missing in the 1AS lines, the 6 kb was missing in the 1BS line and the 12 kb band was missing in the 1DS lines (Figure 4) which is consistent with a gene on the long arms of the group I chromosomes.

#### *SSIV *mRNA expression

**Figure 5:**
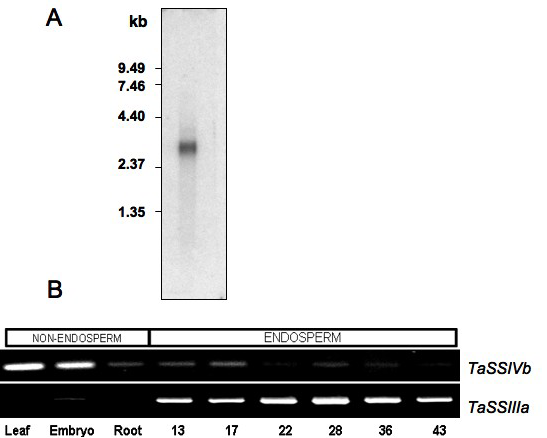
**Transcript size and spatial and temporal expression**. **A **Identification of transcript hybridized to a probe made from *TaSSIVb *EST. Northern analysis was performed with 0.5 μg poly (A+) RNA extracted from developing wheat caryopses. The cDNA probe utilised was a 550 bp fragment from a *Pst*I and *EcoR*I digest of clone 1600-bp (Figure 1). A primary band of approximately 3.4 kb can be seen as a well as a faint band of around 3.7 kb. **B **Spatial and temporal expression of wheat SSs by comparative RT-PCR. RNA was extracted from embryos from caryopses at 25 DPA, endosperm (including the aleurone layer) from caryopses of approximately 4–7 DPA (13 mg), 5–10 DPA (17 mg), 10–16 DPA (22 mg), 16–20 DPA (28 mg), 20–25 DPA (35 mg), 26–32 DPA (43 mg) and leaves and roots from 14-day old seedlings. The mean mass in mgs of the caryopses used, i.e. 13, 17, 22, etc., is shown on the figure. The amounts of all products responded linearly to RNA input, number of PCR cycles and primer concentration.

The size of the *TaSSIVb *transcript was determined by northern blotting. A band of approximately 3.4 kb was detected, which is close to the size of the predicted full-length SSIV transcript based on the PCR-amplified fragments. A faint band of higher molecular mass (~3.7 kb) that hybridized to the probe could also be seen (Figure 5A).

The expression pattern of transcripts encoded by *TaSSIVb *was then determined by semi-quantitative comparative reverse transcription (RT)-PCR (Figure 5B). Expression was highest in leaf and embryo and lower in endosperm where it decreased steadily as the organ matured. This result was reproducible over four different experiments. Expression of the *TaSSIIIa *transcript was also compared to that of *TaSSIVb *because of the close phylogenetic relationship between these isoforms (Figure 5B). The *TaSSIIIa *transcript is strongly expressed in the endosperm, but could be detected in leaf (upon long exposure, data not shown) and embryo albeit at significantly lower levels. Similar expression profiles were obtained in rice with *OsSSIVb *and *OsSSIIIa *[38-40].

#### Expression of SSIV leaf transcript by semi-quantitative comparative RT-PCR under different light conditions

**Figure 6:**
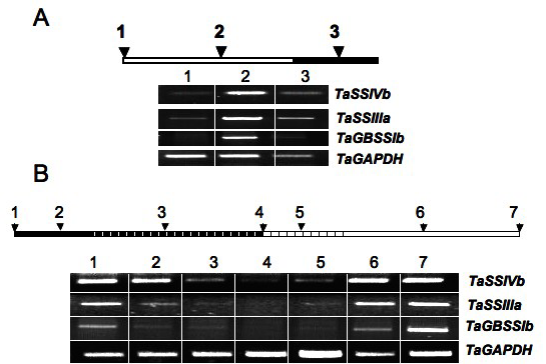
**Transcript analysis of the wheat SSs in leaf by comparative RT-PCR under varying conditions of light**. **A**. Transcript levels of *TaSSIVb*, *TaSSIIIa*, *TaGBSSIb *and *TaGAPDH *during the course of the photoperiod in wheat leaves as determined using comparative RT-PCR. RNA was extracted from leaves sampled from 14-day old wheat plants grown under a regime of 16 h light and 8 h dark in a greenhouse. Open bars correspond to the 16 h-light period; the solid bars correspond to the 8 h-dark period. Leaves were harvested at the end of the dark/beginning of the light period (1), the middle of the light period (2) and in the middle of the dark period (3). Results are typical of three experiments. **B**. Transcript levels of the *TaSSIVb*, *TaSSIIIa*, *TaGBSSIb *and *TaGAPDH *during an alternating 24 h-dark and light period. Plants were grown as described in **A **but were transferred to a growth chamber and acclimated to 16:8 light:dark regime for 2 days. RNA was extracted from leaves of eight 14-day old plants after 16 h light (and 0 h dark) (1), 4 h (2), 16 h (3) and 24 h (4) in the dark, followed by 4 h (5), 16 h (6) and 24 h (7) in the light. Solid bars correspond to the dark period; the white stippled areas correspond to what in a 16:8 cycle would have been the light period. Open bars correspond to the light period; the stippled area corresponds to what in a 16:8 cycle would have been the dark period.

Because *TaSSIVb *is preferentially expressed in leaf it is possible that transcript levels could be responsive to changes in the light-dark regimen and that it could be regulated by the circadian clock. *TaGBSSIb *was included in this study for comparison because it was reported to be unresponsive to light when analyzed by northern blotting [41]. The *TaSSIVb*, *TaSSIIIa *and *TaGBSSIb *transcripts (Figures 6A &6B) were all expressed at high levels in response to light, but after 24 h of continuous darkness (at time point 4 in Figure 6B), transcripts were barely detectable. This indicates that in wheat the circadian clock does not regulate expression of these genes since during the extended dark period, mRNA levels do not follow the pattern expected during the 16 h light period.

### B. Global sequence analysis of SS Isoforms

#### Comparative protein modeling

To further our understanding of the wheat SSIVb gene product and SSs in general, the sequences of this, and 31 SSs and 3 GSs from a broad range of species were analysed to identify common as well as unique features that may underscore the basis for conserved functions as well as differences in SS metabolic action *in planta*. To gain insight on the structural features of SSs, we also compared SS sequences specifically to that of *Agt*GS for which a crystal structure is available.

##### 3D Structure of SSIV

**Figure 7:**
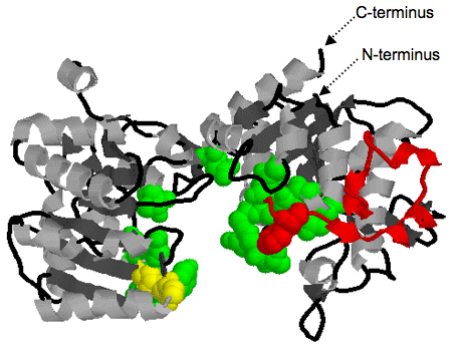
**A proposed structural model for *Ta*SSIVb based on the *Agt*GS structure (Protein Databank code: **1rzu**)**. A putative homology model of the overall protein-fold and secondary structure of *Ta*SSIVb was constructed using T.I.TO: Tool for Incremental Threading Optimization [73,74]. The N- and C-termini are indicated, the two different Rossmann-like α-β-α domains are apparent, as is the large cleft that separates them and makes up the active site. Residues identified as crucial to SS activity are highlighted (green balls) and are outlined in Table 2 and 5. Also highlighted are the residues of the 380s Loop (in red) and the G-X-G motif (in yellow).

A homology model for the C-terminus (amino acids 406–914) of *Ta*SSIVb was built by threading its sequence onto the 3D structure of *Agt*GS (Figure 7). The resulting model is in good agreement with those developed for *Agt*GS and *At*SSIII [26,42]. The two different α-β-α Rossmann-domains of the GT-B family are apparent as is the large cleft which contains the ADP-binding pocket towards the C-terminal side [43]. Residues that take part in catalysis and/or substrate-binding depicted on the model are elaborated in Table 1, which shows amino acids that are conserved across GS and SSs, and Table 2, which highlights those amino acids that are not highly conserved. These residues were identified based on published studies on *Agt*GS and, to a lesser extent, based on site mutagenesis studies of *Ec*GS [18,19,44]. These amino acids are all brought into close proximity of the ADP molecule in response to the predicted change in conformation of the enzyme in going from an opened, relaxed form to a closed state [43].

**Table 1:** Amino acid residues that directly bind the ADPglucose substrate in *Agt*GS and their corresponding invariant residues in plant SS proteins

	**SSs: Group A**			**SSs: Group B**	
***Agt*GS **	**GBSSI**	**SSI**	**SSII**	**SSIII**	**SSIV**

Lys15	Lys91	Lys153	Lys322	Lys1194	Lys435
Gly18	Gly94	Gly156	Gly325	Gly1197	Gly438
Asp138	Asp229	Asp285	Asp447	Asp1309	Asp558
His163	His258	His314	His476	His1337	His586
Asn246	Asn347	Asn408	Asn562	Asn1385	Asn671
Ile297	Val/Ile400	Ile460	Ile619	Val/Ile1437	Val/Ile724
Arg299	Arg402	Arg462	Arg621	Arg1439	Arg726
Gly327	Gly431	Gly490	Gly649	Gly1467	Gly754
Tyr354	Phe458	Phe517	Phe676	Phe/Tyr1499	Tyr784
Asn355	Ser/Asn459	Ser/Asn518	Ser677	Asp1500	Ap785

**Figure 8:**
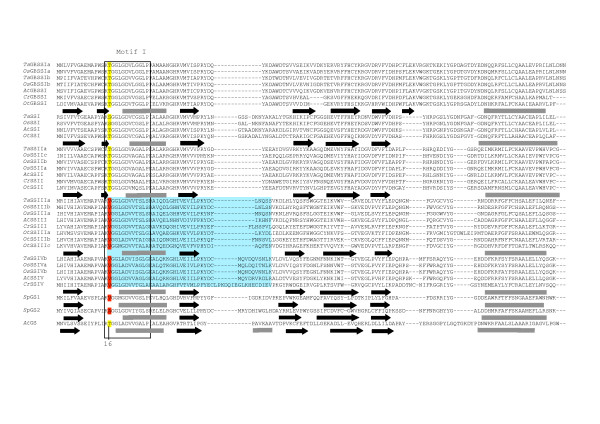
**Prediction of the structural relationship between the wheat SSs and query proteins with the 3D structure of *Agrobacterium tumefacien *glycogen synthase**. The predicted amino acid sequence of 32 SSs from wheat, rice, *Arabidopsis*, *Chlamydomonas *and *Ostreococcus *and two GSs from *Synechocystis *were compared to *Agt*GS. Sequences were aligned using Clustal W [34]. The alignment was manually adjusted using secondary structure predictions. The key is as follows: regions predicted to form α-helices – grey rectangles; black arrows – β-sheets. Conserved amino acid residues are highlighted. The two major Loops, the 380s Loop or stretch of polypeptides (labeled), as well as the second Loop in Motif VI (which is highlighted in green), are indicated.

Amino acids that are part of the catalytic site in *Agt*GS were identified in previous studies [18,19,43,44] and the equivalent residues in plant SSs were identified after alignment of *Agt*GS using ClustalW [34]. Only *Agt*GS amino acids that were conserved across all of the SS isoforms are shown. Data was extracted from the alignment shown in Figure 8.

**Table 2:** Amino acid residues within the substrate binding/catalytic sites in SSs that vary from the corresponding residue in *Agt*GS

	**SSs: Group A**			**SSs: Group B**	
***Agt*GS **	**GBSSI**	**SSI**	**SSII**	**SSIII**	**SSIV**

Thr16	Thr92	Ser/Thr154	Thr323	**Val1195**	**Val436**
Gln140	**His231**	**His287**	His449*	Ser1311①	Gln560
Ile162	Ile257	Ile313	Ile475	Ile1336	Cys585②
Phe167	Tyr/Phe262	**His-318**	**His480**	Tyr/Phe1341	Tyr590②
Ile297	Val/Ile400	Ile460	Ile619	Val/Ile1437	Val/Ile724
Ser298	Gly401	Gly461	Gly620	**Ser/Thr1438**	**Ser/Thr725**
Gly329	Gly433	Gly492	Gly651	**Ala1469**	**Ser756②**
Gly353	Arg/Lys457	Gly516	Gly675	Arg/Lys/Tyr/Thr1498	Lys783②
Asn355	Ser/Asn459	Ser/Asn518	Ser677	Asp1500	Asp785②
Ser359	Ala463	Ala/Ser522	Ala681	Ser1504	Ser789②
Thr381	**Ile485**	Asn554	Asn703	Ser/Thr1526	Thr811

Amino acids identified in *Agt*GS as part of the ADPglucose pocket and are involved in binding and/or catalysis that were not conserved across all of the SS isoforms are shown. Non-conservative amino acids changes that could affect enzyme properties are emboldened. Data was extracted from the alignment shown in Figure 8. Species with an amino acid at the residue other than that in the table are indicated as follows: ①*Ot*SSIIIa/c where the residue is a glutamine. ②*Cr*SSIV. *OsSSIIa – Tyr

##### Analysis of the conserved domains

The length and homology of the conserved starch catalytic (GT-5) and the glycosyltransferase-1 (GT-1) domains of some SSs and GSs were compared to those found in prokaryotic GSs for which a crystal structure exists. The GT-5 domain (Pfam PF08323) was similar among the different SS and GS isoforms with the range of Expect values (E) from 6.5e^-10 ^to 2.5e^-140 ^compared to that of *Agt*GS (data not shown). However there was less similarity in the GT-1 region (Pfam PF00534), with the Group B SSs differing most. E-values were 0.5 to 4.2e^-3 ^in the Group B SSs and from 2e^-7 ^to 4.8e^-16 ^in the Group A SSs when compared to that in *Ec*GS.

##### SS secondary structure

**Figure 9:**
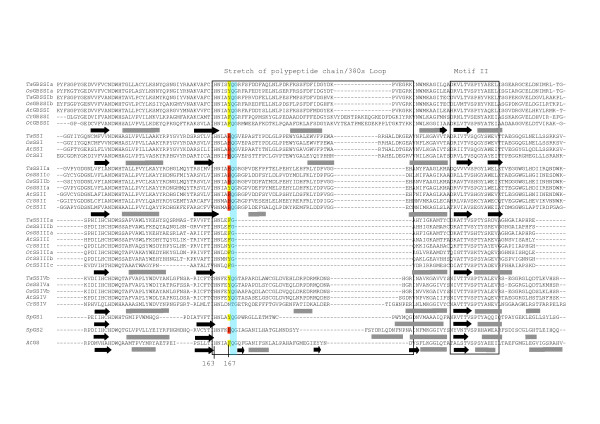
**Prediction of the structural relationship between the wheat SSs and query proteins with the 3D structure of *Agrobacterium tumefacien *glycogen synthase**. The predicted amino acid sequence of 32 SSs from wheat, rice, *Arabidopsis*, *Chlamydomonas *and *Ostreococcus *and two GSs from *Synechocystis *were compared to *Agt*GS. Sequences were aligned using Clustal W [34]. The alignment was manually adjusted using secondary structure predictions. The key is as follows: regions predicted to form α-helices – grey rectangles; black arrows – β-sheets. Conserved amino acid residues are highlighted. The two major Loops, the 380s Loop or stretch of polypeptides (labeled), as well as the second Loop in Motif VI (which is highlighted in green), are indicated.

**Figure 10:**
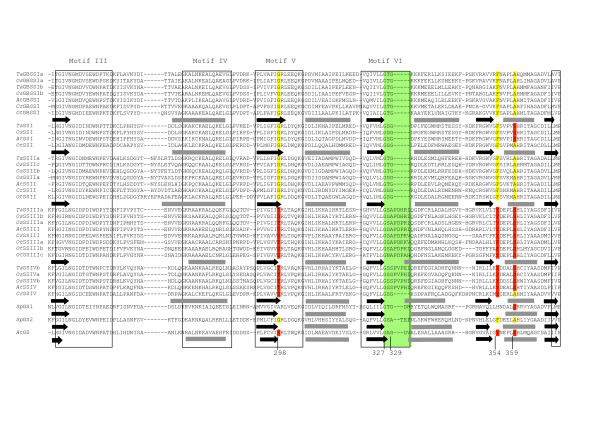
**Prediction of the structural relationship between the wheat SSs and query proteins with the 3D structure of *Agrobacterium tumefacien *glycogen synthase**. The predicted amino acid sequence of 32 SSs from wheat, rice, *Arabidopsis*, *Chlamydomonas *and *Ostreococcus *and two GSs from *Synechocystis *were compared to *Agt*GS. Sequences were aligned using Clustal W [34]. The alignment was manually adjusted using secondary structure predictions. The key is as follows: regions predicted to form α-helices – grey rectangles; black arrows – β-sheets. Conserved amino acid residues are highlighted. The two major Loops, the 380s Loop or stretch of polypeptides (labeled), as well as the second Loop in Motif VI (which is highlighted in green), are indicated.

**Figure 11:**
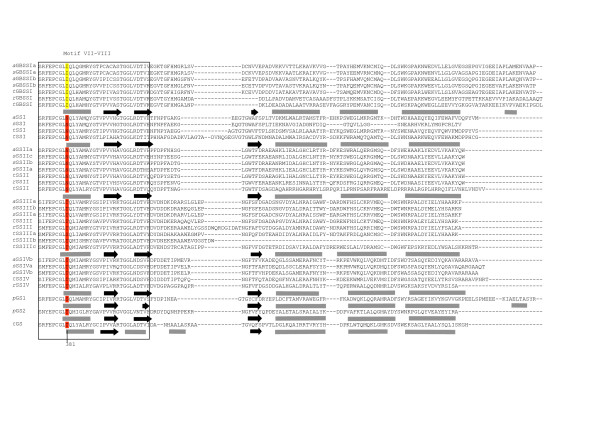
**Prediction of the structural relationship between the wheat SSs and query proteins with the 3D structure of *Agrobacterium tumefacien *glycogen synthase**. The predicted amino acid sequence of 32 SSs from wheat, rice, *Arabidopsis*, *Chlamydomonas *and *Ostreococcus *and two GSs from *Synechocystis *were compared to *Agt*GS. Sequences were aligned using Clustal W [34]. The alignment was manually adjusted using secondary structure predictions. The key is as follows: regions predicted to form α-helices – grey rectangles; black arrows – β-sheets. Conserved amino acid residues are highlighted. The two major Loops, the 380s Loop or stretch of polypeptides (labeled), as well as the second Loop in Motif VI (which is highlighted in green), are indicated.

The secondary structures of the different wheat SSs were compared to *Agt*GS using the Jnet prediction program [45,46]. Some structural features specific to each SS sub-family among the different species could be identified. For example, an additional β-strand is found between β1 and α1 in the Group A SSs that is absent in the *Sp*GSs, *Agt*GS and the Group B SSs, (Figures 8, 9, 10, 11). The hydrophilic "tail" region at the end of the C-terminus that is unique to GBSSI proteins [15] was found to adopt a helical structure. This helical region is also found in *Sp*GS1 but not in *Sp*GS2 (Figure 11).

There are two Loops containing important residues (H163 and Gly329, Table 1; discussed below) that have different lengths in the SSs sub-families. The first Loop, referred to as the 380s Loop [42,43] (and see Figures 7 and 9), is a stretch of 35 amino acids containing a small antiparallel sheet (β8-β9-β10) and one helix (α5) in *Agt*GS but only one predicted helix in the SSs: GBSSI, SSI, SSII and SSIV. In the wheat SSIII, this polypeptide stretch is reduced to 7 amino acids and does not include a helix. This region is highly variable at all levels (nucleotides, amino acids, secondary structure) among the SSs sub-families. The second Loop is located in motif VI, which contains Gly329 in *Agt*GS, and is shown in green on Figures 7 and 10. It is longer in SSIII and SSIV than in the Group A SSs and presents few homologies when compared to *Agt*GS.

##### Active site residues

Amino acid residues that make up the highly conserved regions of SSs and GSs initially found by Gao *et al*., (1998) [27] may now be extended as more SS and GS sequences are available. Residues involved in ADPglucose and/or glycogen/starch binding have also been identified in *Agt*GS which could also be instructive in identifying those defining amino acids needed for catalysis [43] (Figures 8, 9, 10, 11).

The variable amino acid in the Lys-X-Gly-Gly-Leu motif in the SS and GS sequences was first examined. This motif, which occurs at the highly conserved Lys15 in *Agt*GS, has long been considered as part of the ADPglucose active site in SSs and GSs [20,21]. Although this hypothesis has since been brought into question [23], our observations show a high level of intra-group conservation at this residue. It is a valine (non-polar) in Group B SSs, and a threonine or a serine (both polar) in the Group A SSs (Table 2; Figure 8). This appeared true in all 5 SS classes and for all 33 species compared (data not shown). Interestingly, like the Group B SSs, *Sp*GS1 and *Sp*GS2 both have non-polar residues (valine and alanine, respectively) at this site.

Variation in other amino acids believed to play a role in ADPglucose binding in *Agt*GS, may indicate specificities that help define the individual SS classes (Table 2). For example, in *Agt*GS, van der Waals forces among Ser298, Ser359 and Tyr354 are thought to stabilize the adenine heterocycle when ADPglucose binds the enzyme [43]. In the Group A SSs, glycine (aliphatic) is substituted for the polar Ser298 in Motif V (Figure 10). It has also been predicted that the hydroxyl (-OH) side chain of threonine at residue 381 (Thr381) would form a hydrogen bond with the O_2 _atom of the ribose moiety of ADPglucose [43]. However, in all of the GBSSIs analyzed, Thr381 is replaced by isoleucine, which has an aliphatic hydrophobic side chain and cannot form hydrogen or ionic bonds with other groups. Members of the GT5 family have two highly conserved glycines (Gly327 and Gly329 in *Agt*GS) in Motif VI (Figure 10). Interestingly, the G-X-G motif is not conserved in the GT3 family that uses UDPglucose [43]. The Gly329 residue is replaced by serine (polar) in the SSIV sequences and such a substitution would likely affect the functional properties of the protein. In *Agt*GS, His163 and Phe167 are involved in glycogen binding [43] and would be predicted to be found in the GT-5 domain of SSs. However, a previous study stated that His163 is not present in SSs [42]. Our analysis shows that His163 is indeed present and highly conserved among SSs (Figure 9 and Table 2). In contrast, the equivalent Phe167 residue in the SSI and SSII sequences examined are basic (His318 and His380, respectively) and not the aromatic amino acids found in *Agt*GS, the Group B SSs and GBSSIs (Figure 9, Table 2). The only exception we found within the SSII family was *OsSSIIa*, where the conserved tyrosine (aromatic) is evident.

##### Relationship between genomic organisation and location of key amino acids

It has been suggested that if the splicing site of a particular intron coincides at the same amino acid position among members of a group of orthologous proteins, then there is ancient relatedness and conservation of functionality for that amino acid residue [47-50]. The basis for this idea is that proteins are built up through modular exonic segments spliced together using introns and if an amino acid serves an important or critical functional role, these residues will remain relatively unaffected by evolutionary changes in splicing and/or exon re-arrangements.

**Figure 12:**
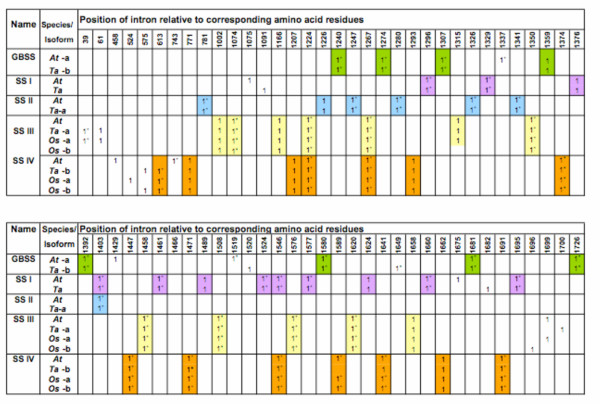
**An alignment showing the position of introns relative to amino acid sequence within SS classes**. An alignment was made with the SS protein sequences by BLASTP [64] and this was compared to the genomic sequence of each protein in turn. The presence of an intron at the position of an amino acid in the alignment is illustrated by a "1" when the splicing occurs inside the amino acid or by "1+" when the splicing occurs just after the amino acid. When an intron was positioned at the same amino acid in all of the sequences of a particular SS subfamily in the alignment, this was illustrated in the figure by color-filled squares. Introns were identified by comparing the cDNA against the genomic sequence of each SS using Spidey at NCBI [75] and were manually checked for non-canonical sites. Genbank Accession numbers ascribed to the nucleotide sequences are as follows: *At*GBSSI [AT1G32900.1A], *TaGBSSIa *[AB019624], *AtSSI *[AF121673]; *TaSSI *[AF091802]; *AtSSII *[AC008261]; *TaSSIIa *[AY133248]. Genbank Accession numbers for the nucleotide sequences of other SSs and amino acid sequences are given elsewhere.

The positions of introns relative to the positions of specific amino acids were compared within the SS subfamilies to look for evidence of evolutionary conservation. In most cases, these positions are conserved among orthologous SS isoforms (Figure 12). However, two consecutive intron positions are conserved among all Group B SSs, and one position is identical for the SSI and SSII sequences examined (Figure 9). Both of these occurrences are within regions of interest within the predicted amino acid sequence. The conserved glutamine identical in SSI and SSII is within the 380s Loop between Motifs I and II (Figure 12), and the consecutive positions for SSIII and SSIV (Group B SSs) occurs within Motif I at the variable residue in the K-X-G-G-L motif between the lysine and valine residues (Figure 8). The amino acid sequences surrounding the conserved splicing positions are shown in blue boxes in Figures 8 and 9.

## Discussion

The first aim of this work was to characterise a cDNA clone with high homology to SSs. The *TaSSIVb *cDNA is related to the SS group of enzymes by the presence of conserved amino acid motifs of the α-glycosyltransferase family that are found within its predicted amino acid sequence. A protein with this activity could be detected, using western blotting and an enzyme assay, when the cDNA was expressed in *E. coli *(data not shown). The wheat SSIV *in vitro *expression was weak (1.5 to 2.3-fold over background; See additional file: "SSIV construct synthesis and *E. coli *expression.doc") possibly because the protein required some component for activity that is not present in the assay as is the case for GBSSI, for example, which is not detected when expressed in *E. coli *because it must be bound to the starch granule to be active [51]. A transcript of similar size to that predicted by the cDNA sequence was detected by Northern blotting. In addition, a weakly hybridizing band of about 3.7 kb could also be seen (Figure 5A), which may be the duplicated *TaSSIVa *gene.

### Gene organisation and chromosomal location

The intron-exon arrangement of the *TaSSIVb *genomic clone isolated (Figure 3) was similar to orthologous genes from rice and *Arabidopsis*. Of interest was the sequence of intron-4 which was highly similar between orthologues. This intron is in a region that when spliced and translated, produces the SSIV-specific N-terminal region. Introns are generally neutral to selection and undergo rapid change during evolution [49], therefore high sequence similarity between orthologous introns indicate a functional constraint during evolution [52].

The chromosomal location of *TaSSIVb *was also determined. It is positioned on the long arms of homeologous group I chromosomes and is syntenic with the *OsSSIVb *gene which maps to Chromosome 5. This differs from the location of many of the genes involved in starch biosynthesis in wheat which co-localise with homeologous group VII chromosomes [36] and to a lesser extent the group II chromosomes [41]. In contrast, *TaSSIVb *and *TaSSIIIa *are on chromosome I, perhaps highlighting the distinct evolutionary origin of these genes as compared to the other SSs [53]. However, *TaSSIVb *and *TaSSIIIa *map to opposite arms of chromosome I therefore chromosomal location offers few clues as to the evolutionary history of these two genes relative to each other.

### Expression analysis

The expression of *TaSSIVb *mRNA was highest in non-endosperm tissue (Figure 5B) and showed fewer marked changes in response to varying light-dark conditions as compared to *TaSSIIIa *(Figure 6B). The *TaSSIVb *gene may be classified as a "steady expresser" [39]. If protein amount and activity reflects message levels then the *TaSSIVb *has a housekeeping role in starch biosynthesis in wheat endosperm as its transcript accumulation levels did not coincide with the period of high carbon flux to starch in the endosperm.

A circadian clock does not regulate *TaSSIVb *or *TaSSIIIa *transcript levels (Figure 6B). The lack of appropriate elements in the promoter regions of the rice homologues *OsSSIVb *and *OsSSIIIa *support this result. Induction of expression of *TaSSIIIa *and *TaSSIVb *during the light period may be a response to sugar accumulation as observed in *IbGBSSI *and *OsGBSSIb *[54,55]. In our hands, *TaGBSSIb *was not circadian clock-regulated which is at odds with the expected expression pattern based on the presence of the relevant cis-element in the *OsGBSSIa *homologue. However, Vrintren *et al*. [39] reported that *TaGBSSIb *expression was not only independent of a circadian clock, but also non-responsive to light.

### Sequence analysis

The second aim in this work was to advance existing knowledge of structure-function relation of starch synthases including the SSIV gene cloned. Known motifs in *Agt*GS predicted to play a role in catalysis and/or binding were compared with SSs analysed. If these sequences are invariant among SSs from diverse species, this may indicate that the corresponding amino acids may function similarly, as conserved sequence motifs suggests an evolutionary relationship between proteins. Structural analysis and alignment of the predicted amino acid sequences of SS and GSs revealed several amino acids potentially crucial in determining specific SS activity. These residues are presented in Table 1. By extension, amino acids within conserved motifs that are distinct between SS isoforms may at least be partially explained, by the important differences in functionality. These will be discussed in turn.

#### GBSSI

GBSSI is the only SS that makes amylose, but it can elongate amylopectin processively if it is bound to this polymer [14,56]. Although the C-terminal "tail" present in GBSSI is unique to this isoform among SSs, it may only define GBSSI's ability to bind to amylopectin [15]. The only other difference that singled out GBSSI compared to other SSs was a Thr381 → Ile amino acid substitution in the active site that was present universally among GBSSI proteins examined. Since this residue interacts with the hydroxyl group of ADPglucose it is easy to imagine that the aliphatic residue will not bind the glucose moiety of ADPglucose in the same way as in *Agt*GS and the other SSs, which have polar residues at this site. This non-conservative substitution would affect the interaction of protein with substrate and explain the low K_m _of GBSSI for ADPglucose and/or the ability of the enzyme to processively elongate maltooligosaccharides [51]. This hypothesis is worth testing using more direct methods such as site-directed mutagenesis.

#### SSI and SSII

The Phe167 residue in the 380s Loop is conserved in all SSs except the SSIs and SSIIs where it is a basic residue and not an aromatic amino acid found in the other SSs. The only exception among SSIIs was the tyrosine found in *Os*SSIIa. The conserved aromatic residue at this position in the GT5 superfamily stacks against the pyranose ring of the glucose molecule of ADPglucose in *Agt*GS [43]. The substitution of a polar residue at this site in SSIs and most SSIIs may affect their ADPglucose-binding ability as compared to *Agt*GS and the other SS classes. The significance of the 380s Loop in SS proteins is unknown, however our findings make a case for a further examination of the role of this region in determining SS activity. This is because the other difference of note between SSI and SSII proteins and all other SSs is the identical intron-amino acid positioning at Glu168 conserved within these isoforms and not shared by the others. This glutamine residue may thus have faced strong selective pressure to be conserved in both SSIs and SSIIs. Furthermore, Glu168 lies immediately adjacent to the histidine which substitutes, again, only in SSIs and SSIIs, for the otherwise highly conserved Phe167 in *Atg*GS. Collectively these data implicate this region of the 380s Loop as potentially holding some functional significance in delineating SS activity, especially for SSIs and SSIIs.

#### Group A vs. Group B SSs

The apparent divergence of these α-glycosyltransferases can in part be explained by differences in the length and sequence homology of their GT-1 domains, substitutions in key amino acid residues and differences in secondary structure near or at the active/binding site that would lead to different spatio-conformational outcomes and possibly change interaction of protein with substrate. The GT-1 domains of the Group A SSs are identical in length while there is little conservation of GT-1 length among Group B SSs. The GT-1 domain interacts with the nucleotide sugars and differences in sequence homology in this region could produce different 3D-folds and variation in reaction mechanisms. Group B SSs also differ structurally from Group A SSs in the G-X-G Loop near β-15 that faces the nucleotide-binding cleft. This motif and Loop was previously believed to be absolutely conserved in all GT5 enzymes, [43] including the Group A SSs, however, it has diverged in the Group B SSs. In SSIVs there is a non-conservative Gly → Ser substitution and, since this is the only difference that distinguishes SSIVs from Group A SSs and SSIIIs, we suggest that this region may help define the unique activity of SSIVs and perhaps, by extension, their role in granule priming. Our conclusions differ from those drawn by Busi *et al*. [42] who inferred that the G-X-G motif is absent in plants SSs; however, their analysis included only *At*SSIII and no other plant SSs.

Within the region that is predicted to directly bind the substrate there are further distinctions between the two main SS groups. In the Group A SSs there is a non-conservative substitution within Motif V of glycine for serine that could alter the symmetry and spatial conformation of the binding site. Furthermore, there is an additional β-strand in the Group A SSs within Motif I which contains the conserved lysine of the K-X-G-G-L. β-strands have an extended conformation which promotes a more linear topography within the secondary structure of the proteins. This additional strand may, again, allow the Group A synthases to interact in a different way with the starch granule and/or substrate and could affect primer preference of these enzymes.

The identity of the variable residue in the K-X-G-G-L motif appeared to be dependent on whether the SS examined was classified as a Group A or B SS. The K-X-G-G-L motif is strictly conserved in all SSs and GSs and has been considered the ADPglucose active site in GSs and SSs [20,21]. Analysis of the crystal structure of *Agt*GS with and without ADPglucose confirms that Lys15 in this motif is involved in substrate binding [43]. Gao *et al*. (2004) put forward the idea that this amino acid may be a determinant of SS chain elongation specificity in plant SSs. Indeed, a major difference between the SSs sub-families is the preference for a primer of a specific chain length; thus, further scrutiny of differences in the sequence of this motif is warranted. Within the highly conserved K-X-G-G-L motif, the variable residue is non-polar in the Group B SSs and polar in the Group A SSs (Figure 8). Another essential consideration is the conservation of intron position with respect to the valine amino acid residue within motif I in the Group B SSs, highlighting, perhaps, that this valine residue is fundamental to the Group B SSs' metabolic action as there is evidence of strong evolutionary selective pressure for its conservation (Figure 9).

A distinguishing feature that separates SSI and SSII of the Group A SSs from the Group B SSs and GBSSI is a conserved glutamine within the 380s Loop. As previously noted [42,43], the 380s Loop is highly variable in length among the different GSs and SSs. It is therefore probable that this Loop is not crucial for SS global activity but it could still confer specificity to individual SSs. The 380s Loop supports communication between the glycogen-binding and catalytic sites of *E. coli *maltodextrin and glycogen phosphorylase with an ensuing spatio-conformational change after substrate-binding ([42] and references therein). A role for this region in SSs or GSs has not yet been determined. Elements found in the equivalent region in glycogen phosphorylases necessary for binding to a carbohydrate are lacking in SSs and GSs [43]. This raises questions about the role of the 380s Loop in SSs. Still, intron-amino acid positioning suggests that the Glu168 residue in this region is highly conserved in SSIs and SSIIs, which points some functional significance of this amino acid and region to SS enzymatic action.

### Proposed role for 380s Loop

There is evidence to suggest that some SSs can catalyse the initiation and elongation of glucan chains. *Agt*GS is able to prime glycogen synthesis independent of a glycogenin protein and Roldan *et al*. [11] drew a comparison between the priming functions of *Agt*GS protein and that of SSIV in *Arabidopsis *based on the phenotype of the *At*SSIV mutant. Indeed, some SSs are able to prime starch synthesis *in vitro *[28]. To explain this possible self-priming function a 2-point insertion mechanism was elaborated by Guan & Keeling [57] whereby an SS would bind two ADPglucose molecules at different sites and α-1,4-glucan chain synthesis would occur *de novo *by the transfer of the glucose moiety of one ADPglucose to the other. Considering the position of Lys15 and the conserved Gly18 in Motif, I far from the catalytic core of the enzyme, it is initially difficult to see how they could determine primer specificity. However, these residues are invariant in the SS/GS family, suggestive of a functional role. One possibility is that after ADPglucose binding at the active site within the cleft or ADPglucose-binding pocket, the closed structure which results brings the α-1 and β-1 sheets containing the K-X-G-G-L motif and any primer it binds, e.g. another ADPglucose molecule or glucan chain, into proximity of the active site. If the 380s Loop functions in SSs in the way proposed for phosphorylases [42,58-60], then the movement of this Loop during the open-close transition after ADPglucose binding may be an important part of the mechanism that drives the specificity of action of the SSs within the catalytic region. This mechanism would not require that the 380s Loop directly bind a carbohydrate. The only region that is consistently different among all SSs is the 380s Loop and these differences may possibly be translated, although indirectly, into different interactions with substrate and/or glucan primer.

Although it is suggested here that the 380s Loop could have a role in determining SS specificity, it does not rule out the possibility that other regions of SSs may also contribute or may be more important for SS functionality. In this study we focused our comparisons exclusively on the two-lobes of the C-terminus containing the GT1 and GT5 domains. It has been established that this region is responsible for catalytic activity and also, differences in substrate preference, affinity and reaction velocity between SSs [15,23,24]. However the function of the N-terminus in potentially defining SS activity is not yet known. This region may help to confer SS unique action by influencing or facilitating their interaction with other starch biosynthetic enzymes and/or substrate [29,30]. The formation of starch biosynthetic enzyme complexes especially could modify the activity of each SS isoform and their relative contribution to starch glucan assembly.

### Evolutionary relationship between cyanobacterial GSs and SSs

Analysis of algal SSs and cyanobacterial GS proved informative in helping to understand the function and relationship between the classes of these enzymes. Of particular interest, *Ostreococcus tauri *does not have SSIV but three isoforms of SSIII [6]. Granule initiation does not seem to be essential in *Ostreococcus *as this alga partitions its starch granules from mother to daughter cells by binary fission [6]. If SSIV is part of the biological mechanism involved in granule initiation [11], it is also possible that *Ot*SSIIIa performs a similar function. Sequence analysis of *Synechocystis *PCC 6803 GSs also clarified our view of SS relationships. *Synechocystis *has two forms of GS, one of which, *Sp*GS2, is closely related to the Group B SSs while the other, *Sp*GS1, is more similar to the Group A SSs (Figure 2). Interestingly, the C-terminal extension found only in GBSSI polypeptides is also present in *Sp*GS1. This raises intriguing possibilities about the evolutionary relationship between *Sp*GS1 and the GBSSIs, and, when it is considered that all members of the Group A SSs have the ability to bind to the starch granule, the functional association of *Sp*GS1 with the Group A SSs as a whole.

Phylogenetic analysis of the origins of starch biosynthetic genes in higher plants and green and red algae suggests there to be a complex set of evolutionary events with vertical and lateral gene transfers between host and endosymbiont cells [35]. In spite of this it is widely accepted that genes from a cyanobacterial endosymbiont are now present in plant host nuclei [61,62] and some of these genes have retained much of their sequence identity. The clear separation of GSs into two distinct clades, and sequence similarities between them and SSs from one group or the other, suggests that the Group A and Group B SSs evolved directly from the two independent GS types. The SSIV group could have evolved from *Sp*GS2, and subsequent gene duplication events might have led to the evolution of SSIIIs. One exon each in the wheat and *Arabidopsis *Class III SSs (exons III and I, respectively), contains a significant proportion of the coding region that is present in SSIII but not SSIV or other SSs [53]. It is possible that the Class III SSs arose as a result of duplication of the SSIV followed by a single insertion event of a gene sequence represented by these exons. The resulting difference in gene structure, as well as subsequent changes selected for during evolution, might have led to two distinct gene products that are sufficiently diverged to constitute two different gene classes. High sequence similarity of intronic sequences and intron-amino acid positions is supportive of the notion that SSIII and SSIV followed a common evolutionary path.

## Conclusion

The basic cloning and characterisation of *TaSSIVb *showed that the SS it encodes is similar to other SSIVs and has almost 50% similarity to prokaryotic GSs and higher plant SSIIIs. Phylogenetic and sequence analysis suggests that SSIs, SSIIs and GBSSIs have a distinct evolutionary origins as compared to SSIIIs and SSIVs. This bifurcation is supported by differences in secondary structure and amino acid residue substitutions in what are accepted to be critical and/or conserved regions for ADPglucose binding and/or catalysis. The 380s Loop is proposed to be potentially a determinant of SS-specificity and through its involvement in the open-closed transition of the active site during ADPglucose binding as has been suggested for glycogen phosphorylases. The valine residue within the highly conserved K-X-G-G-L motif appears to have faced strong evolutionary selection in SSIII and SSIVs and it may affect primer/substrate binding of these SSs compared to SSIs, SSIIs and GBSSIs. SSIII and SSIV also differ from other SSs at the highly conserved G-X-G motif near the nucleotide-binding cleft. We stress that although these predictions of structure-function characteristics of SSs must be confirmed by experimental data, they provide a useful framework for designing an in-depth investigation of the role of each SS in the construction of the 3D architecture of starch. Such studies can help with efforts to engineer starch with modified crystalline structure and physico-chemical properties.

## Methods

### Plant material

Wheat (*Triticum aestivum *L. cv. Hi-Line) plants were grown in a greenhouse at a minimum temperature of 15°C, with supplemented lighting to give a photoperiod of 16 h during the winter. Genomic DNA of Chinese Spring nullisomic-tetrasomic lines, were a kind gift from Dr. Petra Wolters (DuPont-Pioneer, Crop Genetics) and Dr. David Benscher (Department of Plant Breeding, Cornell University). We thank Prof. Jon Raupp (Kansas State University) for seeds of Chinese Spring ditelosomic lines.

### Materials

ADP-[U-^14^C] glucose was purchased from Amersham Pharmacia (Piscataway, NJ). Dowex 1 × 8 resin, ADPglucose and amylopectin were purchased from Sigma (St. Louis, Missouri).

### RNA extraction

RNA was extracted from various tissues of wheat using TRIzol reagent (Invitrogen, Carlsbad, CA). Tissues included embryos from caryopses harvested at 25 DPA, endosperm (including the aleurone layer) from caryopses harvested at approximately 4–7 DPA, 5–10 DPA, 10–16 DPA, 16–20 DPA, 20–25 DPA, 26–32 DPA, whole caryopses harvested at 3, 7 and 14 DPA, and leaves, roots and the aerial parts of 14-day seedlings. Poly (A)+ RNA was purified from total RNA using Pharmacia QuickPrep RNA purification kit (Amersham Pharmacia Biotech, Inc., Piscataway, NJ).

### cDNA library construction and EST analysis

First-strand cDNA synthesis was catalysed by Superscript II Reverse Transcriptase (Invitrogen, Carlsbad, CA) with a nucleotide mixture containing 5-methyl dCTP. The cDNA library was ligated 5'→3' into an *EcoRI/Xho I*-digested pBluescript SK+ vector according to the manufacturer's protocol (Stratagene, Cedar Creek, TX) and maintained in *Escherichia coli *DH10B cells (Life Technologies, Carlsbad, CA). The SSIV EST [Genbank:BT009276] was identified by sequencing randomly picked bacterial colonies from a developing wheat seedling cDNA library made using a previously described method [63].

### Generation of the full-length *SSIV *sequence by 5'RACE of wheat cDNA libraries

DNA was prepared from each wheat library using a DNA miniprep kit (Qiagen, Valencia, CA). An aliquot of DNA (1 ng.ul^-1^) was used as a template in separate PCR reactions with 10 μM each of T3 vector primer (5'-GCC AAG CTC GGA ATT AAC CCT CA-3') and a gene-specific primer complementary to the 5' end of EST [Genbank:BT009276]. PCR amplification was done with GC-Advantage polymerase mix (Clontech, Palo Alto, CA). Cycling parameters were 94°C for 1 min; 10 cycles of 94°C for 30 s, 68°C for 30 s, 72°C for 4 min, then 25 cycles of 94°C for 30 s, 63°C for 30 s, 72°C for 4 min, and an extension time of 72°C for 7 min in a PE 9700 Thermocycler (Perkin Elmer, Norwalk, CT). The products of the PCR reaction were diluted 1:100 and the DNA re-amplified using a nested primer and the vector primer SK-long (5'-GCC GCT CTA GAA CTA GTG GAT CCC CCG GGC TGC AGG A-3'). PCR products were gel-purified (Qiaquick, Qiagen, Valencia, CA) and subcloned into pCR 2.1 (TOPO-TA, Invitrogen, Carlsbad, CA) or pGEM-T (Stratagene, Cedar Creek, TX) vectors. At least four transformants containing an insert were selected and sequenced in both directions using an ABI 3700 capillary Sequencer with the ABI BigDye terminator chemistry (The PE Corporation, Foster City, CA). The longest PCR product was used to design primers for additional 5'-RACE. The antisense oligonucleotide primers used in conjunction with a vector primer to amplify contiguous sequences of the entire cDNA were (please refer to Figure 1): for Sequence A (nucleotides 1 to 1023), 5'-GCA GAT CAT GAT TGT GGT CCA ATA-3' and 5'-AGC ATG CTC TAC TTG GTT TGC TG-3'; for Sequence B (nucleotides 991 to 1778), 5'-AAC AAA TGC AGT TTG CCA GTC A-3' and 5'-CTG GAT GCT GTG GCT CAA TAA A-3'; and for Sequence C (nucleotides 1658 to 2194), 5'-TGC AAG GGT TCC ATG TAT CTG TG-3' and 5'-CCC TCT GAG CGA ACC TCT AGT GC-3'. To amplify fragment 1600 bp-RACE, primers 5'-CGC CCA AGC AGA TAC GAT GAA A-3' and 5'-TCA TAC TTC AAA ATC AGC CGG A-3' were used. To amplify fragment 2100 bp-RACE, the primers were identical to those used to amplify Sequence C and the PCR conditions similar except that Advantage polymerase mix was used and the nested PCR was done using a 1:200 dilution of the primary PCR reaction as template.

### Northern and Southern hybridisations

Northern analysis was performed with 0.5 μg poly (A+) RNA extracted from developing wheat caryopses, separated on a 1.4% formamide-formaldehyde agarose gel and blotted onto HyBond N+ (Amersham Pharmacia Biotech, Piscataway, NJ) according to the manufacturer's protocol. The cDNA probe was a 550 bp fragment from a *Pst*I and *EcoR*I digest of clone 1600 bp-RACE in TOPO-TA (Invitrogen, Carlsbad, CA). The probe was labelled using RadPrime DNA Labelling System (Invitrogen, Carlsbad, CA). Hybridisation was done using PerfectHyb Plus solution (Sigma, St. Louis, MO) at 65°C according to the manufacturer's protocol. The membrane was washed twice for 20 min in 2 × SSC and 1% (w/v) SDS at 65°C, and five times for 20 min in 0.1 × SSC and 0.1% (w/v) SDS at 65°C, until the background radioactivity was almost zero. Southern analysis was performed with genomic DNA extracted from leaves of *Triticum aestivum *cv. Chinese Spring nullisomic-tetrasomic and ditelosomic lines using standard techniques. A 20 μg aliquot of each sample was digested with *EcoRV *and resolved on a 0.8% agarose gel. Blotting, hybridisation and washing of the filter was as described for RNA analysis.

### mRNA detection using comparative Reverse Transcription-PCR

Reverse transcription of wheat RNA, (treated with DNase I – RNase-free, Ambion, Austin, TX) was performed with 0.1–1.0 μM of antisense gene-specific oligonucleotide primer (MWGBiotech, High Point, NC) at 42°C for 30 min with an RNA-PCR kit (Roche Molecular Systems, Branchburg, NJ). PCR was done using the reverse transcription reaction as a template and Advantage-2 GC Polymerase mix (Clontech, Palo Alto, CA) with the addition of the sense primer, using the following PCR conditions: *TaSSIIIa *and *TaSSIVb*, 94°C for 30 s, 55°C for 60 s, 72°C for 90 s for 25 cycles and for *TaGAPDH *and *TaGBSSIb*, 94°C for 15 s, 59°C for 30 s, 72°C for 60 s for 22 cycles. Each cycle had a final extension of 72°C for 7 min. All reactions were done in a PE 9700 Thermocycler. The primers used to amplify the *SSIV *transcript were 5'-GATACAAACGGCACTGGACGAA-3' and 5'-CTCCATTTTTAGCTCGCGTTCT-3'. To amplify *TaSSIIIa *[Genbank:AF258608] the primers used were 5'-AATGGGATGTTTGGCGTTGG-3' and 5'-GTATAAGGGACCGGGATAAAA T-3'. To amplify *TaGBSSIb *[Genbank:AF109395] the primers used were 5'-GTTCTGCCTTTTGTGCCTTGCT-3' and 5'-GCAGCATCAGTTCCTCCTCCAT-3. To amplify *TaGAPDH *[Genbank:AF251217] the primers used were 5'-TCTCCAACGCTAGCTGCACCAC-3' and 5'-GGAAGTCAGTGGAAACAAGGTC-3'. Primer pairs were used at a final concentration of 0.2 μM with the exception of the *TaGAPDH *pair, which was 0.1 μM in the PCR reaction. The amounts of all products responded linearly to RNA input and were as follows: Ta*SSIIIa*, 50 ng and for *TaSSIVb, TaGAPDH *and *TaGBSSIb*, 400 ng. Each reaction was repeated at least four times. Ethidium bromide-stained PCR products were visualized with an Eagle-Eye II Imager (Stratagene, La Jolla, CA). Each PCR product was sequenced completely to confirm its identity. A product was not observed in RNase-treated controls, or when RNA was used as a template in a PCR reaction.

### Isolation of genomic DNA

The *SSIV *genomic clone was isolated by using PCR on genomic DNA from *Triticum aestivum *leaves (line N1BT1D) using specific primers designed from the cDNA sequence [Genbank:AY044844]. Two Taq polymerase enzymes (and their corresponding buffers) were used: Expand Long template PCR system (Roche Molecular Systems, Branchbury, NJ) and Advantage-2 GC Polymerase mix GC-2 (Clontech, Palo Alto, CA). Each PCR reaction contained 250 ng genomic DNA, 0.3 mM of each primer and 0.25 U of Taq DNA polymerase. Amplification was done with 1 denaturation cycle at 94°C for 2 min, 30 cycles of 20 sec at 94°C, 30 sec at 62°C and 4 min at 68°C, followed by a final cycle of extension at 68°C for 10 min in a Techne Gradient PCR Cycler (Techne Inc., Burlington, NJ). The PCR reaction was loaded and separated on a 0.8% (w/v) agarose gel. The visualized bands were extracted from the gel and the purified fragments cloned into the TOPO-XL vector (Invitrogen, Carlsbad, CA) and sequenced as described earlier.

### DNA and protein sequence analysis

Computer analyses of all cDNA sequences were done using the program VectorNTI (Invitrogen, Carlsbad, CA). Similarity searches of nucleotide and protein sequences were performed with BLASTP using the non-redundant database at NCBI [64] Sequence alignments were accomplished using ClustalW [34] on the European Bioinformatics Institute's server and subsequent phylogenetic analyses were computed with MEGA3 [65]. The conserved domains within the predicted amino acid sequences were identified using the Pfam database [66] ProDom [67] and Simple Modular Architecture Research Tool (SMART) [68-70]. Prediction of the structural relationship between protein sequences of unknown 3D structure against proteins with a known 3D structure were made using SAM-T02 [71,72]). The Jnet server was used to predict secondary structure [45,46]. Protein models were constructed using T.I.TO: Tool for Incremental Threading Optimization at [73,74].

## Abbreviations

SS: Starch synthase; GS: Glycogen synthase; *Agt*GS: *Agrobacteruim tumesfasciens*; GBSSI: Granule Bound Starch Synthase I; GT5: glycosyltransferase-5; GT3: glycosyltransferase-3.

## Authors' contributions

MSL cloned the genomic sequence, performed the bioinformatics analysis and drafted the manuscript. LDH created the constructs and performed *E. coli *expressions. KEB and DMB conceived of the study. DMB participated in its design and coordination, performed molecular experiments and wrote the manuscript. All authors read and approved the final manuscript.

## Supplementary Material

Additional file 1 **SSIV plasmid construction and expression in an *E. coli *heterologous system.** A detailed description of the cloning and design of different SSIV constructs and their subsequent expression in *E. coli*. Includes Figure 1 – a schematic of the cloning procedure. Click here for file
