# Supplementary material for: Cloning, characterisation and comparative analysis of a starch synthase IV gene in wheat: functional and evolutionary implications
Source: BMC Plant Biol. 2008 Sep 30;8:98. doi: 10.1186/1471-2229-8-98 (PMC2576272; doi:10.1186/1471-2229-8-98)
Supplement: Additional file 1 — SSIV plasmid construction and expression in an E. coli heterologous system. A detailed description of the cloning and design of different SSIV constructs and their subsequent expression in E. coli. Includes Figure 1 – a schematic of the cloning procedure. [file 1471-2229-8-98-S1.doc]

**Description of Additional File 1**

Description of the engineering of different SSIV constructs and their subsequent expression in *E.coli.*

**A. SSIV Plasmid construction**

We assembled the contiguous *SSIV* coding region by routine molecular cloning (See Additional figure 1). The steps in this process were as follows. **(1)** A PCR product was amplified from the 1600bp RACE template using forward primer (SS4-1051) and reverse primer T7. This amplified fragment was placed in pCR4-TOPO digested with *Bam*HI and *Eco*RI and ligated in-frame into pinpoint Xa3 expression vector (Promega, Madison, WI). **(2)** Primer 5’BamHI-ATG-GRIN-EST was designed to correspond to the N-terminus of the EST. This fragment was placed in pCR4-TOPO, digested with *Bam*HI/*Eco*RI and ligated in-frame into pXa3. **(3)** The N-terminal *Bam*HI ATG 1600 fragment was removed from Xa3 with *Bam*HI and *Bsr*GI and ligated in to the *Bam*HI and *Bsr*GI sites in the EST-Xa3 vector fragment. This clone was designated Met-1600-EST-Xa3.

To assemble the full-length contiguous *SSIV* ORF, we used two methods successfully. One method **(A**) used a traditional restriction enzyme and ligation approach. The second method **(B)** used an overlapping fragment and PCR approach. All PCR was conducted with Klentaq or TAKARA (Clontech, Palo Alto, CA) Taq polymerase with proofreading enzyme for high fidelity amplifications. All *SSIV* clones were verified by sequencing.

For the traditional restriction enzyme approach, the N-terminal 2100 RACE fragment was ligated onto the 1600-EST. **(4)** Primers (5’ BamHI MIQ-SS4) and 2100 primer with a TGA was used to PCR-amplify the 2100 in the pGEM-T vector (Promega, Madison, WI) and ligated into pCR4-TOPO vector. The clone, now called MIQ-2100-pCR4-topo, was singly digested with *Pst*1 **(5)**. The *Pst*I vector fragment, containing the *SSIV* N-terminal ORF beginning with MIQ, was re-ligated.

**(6)** The 1600-EST-Xa3 clone was digested with *Pst*1 and the 5’ *Pst*I 1600-EST with stop codon and 3’ *Pst*I insert fragment was ligated into the 2100 *Pst*I pCR4TOPO **(7)**. The full-length *SSIV* ORF in pCR4-topo was cloned into pBSSK using vector *Eco*RI sites **(8)**. A double digest with *Bsr*GI and *Not*I was used to remove a C-terminal insert from the EST-Xa3. This insert was then ligated into the *Bsr*GI and *Not*I sites in the full-length SSIV-pBSSK clone. **(10)** The complete *SSIV*-ORF-BSSK clone would take advantage of the T7 promoter and also the origin of replication, which was compatible with the second plasmid origin of replication for co-transforming into the glgA, BL21 cells.

For the second approach, we used a PCR method to assemble the N-terminal MIQ 2100 fragment to the 1600-EST fragment. Linearized large fragments were first prepared by PCR and gel-purified (Qiaquick, Qiagen, Valencia,CA) **(11)**. Equal molar fragments were combined and denatured at 95°C for 4 minutes then slowly cooled so that overlapping ends would anneal. Takara polymerase (BD Biosciences), dNTP and primers 5’MIQ-2100 and 5’3024-3’ (Refer to Additional figure 1 for primers) were added and PCR performed with 35 cycles (see Additional figure 1). One product that was gel-purified and Qiagen-cleaned contained the full-length *SSIV* ORF. This was cloned into pGEM-T vector **(12)**

**B. Expression of *TaSSIVb* in *E.coli* heterologous system**

To confirm coding potential of the full-length and truncated sequences in the clones, we ligated each *SSIV* fragment into an *E. coli* expression vectors, biotin-tag Xa3 (Promega, Madison, WI) and His-tag pET (Novagen, Madison, WI). The SSIV constructs were co-transformed into *E. coli* with a construct that over-expresses the bacterial ADP-glucose pyrophosphorylase operon [62]. Activities of full-length and truncated *SSIVb* were assessed in wild type BL21(DES) using [^14^C]-ADPG substrate and routine starch synthase activity protocols [38, 48]. To test functionality in *E. coli*, we transformed SSIV constructs into the mutant *glgA-* *E. coli* strain lacking the glycogen synthase operon. We anticipated that constructs with expressed polypeptides containing core regions (2100-1600-EST, EST) would exhibit activity because the SSIV core region is highly conserved.

Biotin-tagged truncated SSIV constructs were transformed into *E.coli* [JM109, BL21(DES)]. Soluble protein extracts were fractionated with SDS-PAGE, transferred to PVDF membrane (Invitrogen, Carlsbad, CA), and screened for presence of the biotin-tagged recombinant SSIV truncated polypeptides with streptavidin alkaline phosphate conjugate and substrate NBT-BCIP. Analyses of blotted proteins revealed SSIV truncated polypeptides (2100, 1600, 1600-EST, EST) of sizes that are consistent with their predicted ORFs.

To detect activity of the SSIV cDNA-pBluescript construct it was transformed into *glgA-* RH98 (a gift from Dr. R. Hengge-Aronis, Department of Biology, University of Konstanz, Germany) and grown in LB broth supplemented with 2% glucose, 2.5 mM betaine and 1M sorbitol. To minimize improper folding into inclusion bodies [77]. Overnight cultures were diluted 1:10 into 200 ml of LB media and were cultured at 37°C with orbital shaking. 1 mM IPTG was added at OD_600_ to induce expression. Soluble extract was prepared by sonication and starch synthase activity was measured as previously described [13]. Protein content was measured as previously described [78]. Activity 1.5-2.3-fold over that of the empty pBluescript vector was observed in five different measurements.
